# First record of *Phlebotomus (Transphlebotomus) mascittii* in Slovakia

**DOI:** 10.1051/parasite/2016061

**Published:** 2016-11-16

**Authors:** Vit Dvorak, Kristyna Hlavackova, Alica Kocisova, Petr Volf

**Affiliations:** 1 Charles University Prague, Faculty of Science, Department of Parasitology Vinicna 7, Prague 2 12844 Czech Republic; 2 University of Veterinary Medicine and Pharmacy, Institute of Parasitology Komenskeho 73 Kosice 04181 Slovakia

**Keywords:** Sand fly, *Phlebotomus mascittii*, Slovakia, *Transphlebotomus*, Northern limit

## Abstract

A large-scale entomological survey was carried out in summer 2016 in the Czech Republic and Slovakia. It revealed, for the first time, the presence of the phlebotomine sand fly *Phlebotomus (Transphlebotomus) mascittii* Grassi, 1908 (Diptera: Phlebotominae) in south-western Slovakia. Species identification of a captured female was confirmed by both morphological and sequencing (COI) analyses.

## Abstract (fr)

Une enquête entomologique à grande échelle a été réalisée durant l'été 2016 en République Tchèque et en Slovaquie. Elle a révélé, pour la première fois, la présence de *Phlebotomus (Transphlebotomus) mascittii* Grassi, 1908 (Diptera : Phlebotominae) dans le sud-ouest de la Slovaquie. Une femelle a été capturée et son identification repose sur des caractères morphologiques et moléculaires (COI).

## Introduction

Phlebotomine sand flies (Diptera: Psychodidae) are vectors of several infectious pathogens including parasitic protozoans of the genus *Leishmania* and phleboviruses and are therefore of great importance in human and veterinary medicine [5, 11]. Although in Europe they occur typically in the Mediterranean countries, some species extend their range of distribution into regions north of their core areas [12]. As the presence of a vector species is one of the risk factors for *Leishmania* transmission [19], it is very important to study the limits of sand fly occurrence because their presence in areas at the edge of their distribution range may be overlooked. This study was conducted as part of the VectorNet project, which focuses on mapping sand fly presence in Europe, including the northern limits of their distribution. To pursue this objective, we surveyed southern parts of the Czech Republic and Slovakia for sand fly presence.

## Materials and methods

A field survey to detect sand flies was conducted from July 6 to July 31, 2016 at 41 localities in south-eastern Slovakia, south-western Slovakia (localities from 9 counties) and southern Moravia, Czech Republic (localities from 2 counties) (Table 1). Moreover, collections of insects from past seasons (2012–15) in the same localities in south-eastern Slovakia, as surveyed in 2016 and stored in ethanol, were inspected under a stereomicroscope. Centers for Disease Control (CDC) light traps (John W. Hock) baited with CO_2_ (dry ice) were placed mostly inside or close to animal shelters and/or organic material both on commercial farms and in private houses where no insecticide spraying was applied. New collection nets from the manufacturer were deployed to exclude possible contamination by sand fly specimens from previous field studies. The traps were set about 2 h before sunset and collected the next morning. Captured insects were killed by freezing in a polystyrene box with dry ice and manually inspected on a sheet of filter paper and under a stereomicroscope.

**Table 1.** Localities surveyed during the entomological survey in the Czech Republic and Slovakia.

Locality	County	Country	Date	No. of traps	Habitat	Potential hosts	N	E	ASL
Trebejov	Košice – okolie	Slovakia	7.7.2016	4	Horse farm	Horses, poultry	48°50′17.00″	21°13′04.00″	234
Kosicke Olsany	Košice – okolie	Slovakia	7.7.2016	5	Village house	Poultry, pigs, rabbits	48°43′55.09″	21°20′30.57″	191
Velaty	Trebišov	Slovakia	8.7.2016	7	Agricultural farm	Poultry, sheep, rabbits	48°31′25.95″	21°38′57.75″	175
Velka Trna	Trebišov	Slovakia	8.7.2016	3	Village house	Poultry	48°27′59.84″	21°40′57.27″	193
Michal’any	Trebišov	Slovakia	8.7.2016	6	Agricultural farm	Sheep, cattle	48°30′44.32″	21°36′55.94″	124
Paňovce	Košice – okolie	Slovakia	9.7.2016	11	Village houses	Poultry pigs, cattle	48°38′58.12″	21°03′38.66″	247
Batka	Rimavská Sobota	Slovakia	10.7.2016	4	Agricultural farm	Cattle	48°22′48.09″	20°09′41.57″	187
Dulovo	Rimavská Sobota	Slovakia	10.7.2016	4	Agricultural farm	Cattle	48°22′33.39″	20°11′27.16″	187
Drna	Rimavská Sobota	Slovakia	11.7.2016	3	Sheep farm	Sheep	48°15′48.01″	20°07′10.93″	186
Chrámec	Rimavská Sobota	Slovakia	11.7.2016	8	Village houses	Poultry, pigs, dogs	48°16′44.95″	20°11′15.45″	167
Dobra	Rimavská Sobota	Slovakia	11.7.2016	5	Agricultural farm	Poultry, pigs, sheep, cattle, dogs	48°19′12.20″	20°06′04.00″	208
Ples	Lučenec	Slovakia	12.7.2016	4	Agricultural farm	Sheep, cattle	48°13′41.83″	19°44′51.30″	224
Lipovany	Lučenec	Slovakia	12.7.2016	3	Agricultural farm	Cattle	48°13′03.43″	19°41′59.43″	216
Mulka	Lučenec	Slovakia	12.7.2016	3	Agricultural farm	Cattle	48°16′26.37″	19°41′59.73″	182
Trebel’ovce	Lučenec	Slovakia	12.7.2016	3	Village houses	Poultry, rabbits, cattle	48°17′02.24″	19°42′50.52″	182
Laza	Lučenec	Slovakia	12.7.2016	3	Village house	Sheep	48°17′39.09″	19°43′07.55″	172
Dubovany	Hlohovec	Slovakia	13.7.2016	3	Village house	Poultry, pigs	48°31′34.98″	17°43′48.33″	160
Šalgočka	Hlohovec	Slovakia	13.7.2016	8	Village houses	Poultry, rabbits, pigs	48°20′10.64″	17°48′39.64″	140
Limbach	Pezinok	Slovakia	14.7., 27.7.2016	3 + 2	Village house	Horses	48°17′33.66″	17°13′12.44″	203
Svätý Jur	Pezinok	Slovakia	27.7.2016	3	Horse farm	Dogs, horses	48°14′43.93″	17°13′08.19″	130
Pernek	Malacky	Slovakia	14.7., 26.7.2016	5 + 7	Agricultural farm	Horses, elephant	48°21′54.59″	17°08′20.97″	257
Jablonové	Malacky	Slovakia	26.7.2016	2	Dog kennel	Dogs	48°21′34.96″	17°04′04.48″	201
			26.7.2016	3	Horse farm	Dogs, horses	48°21′25.40″	17°04′51.48″	199
Lozorno	Malacky	Slovakia	27.7.2016	4	Agricultural farm	Poultry, sheep, goats, pigs	48°19′22.80″	17°04′41.56″	235
Čierna Voda	Senec	Slovakia	27.7.2016	3	Horse farm	Pigs, horses	48°13′28.86″	17°13′46.11″	130
Popice	Znojmo	Czech Rep.	15.7., 30.7.2016	4 + 4	Village house	Goats, horses	48°49′18.10″	16°00′54.20″	287
			30.7.2016	2	Sheep pasture	Sheep	48°49′15.22″	16°00′39.26″	300
Havraniky	Znojmo	Czech Rep.	15.7., 30.7.2016	4 + 2	Village house	Rabbits, horses	48°48′47.07″	16°00′15.11″	310
Naceradice	Znojmo	Czech Rep.	15.7., 30.7.2016	2 + 2	Village house	Poultry	48°49′09.43″	16°06′51.18″	231
Jecmeniste	Znojmo	Czech Rep.	16.7.2016	2	Sandstone quarry	Wild birds and rodents	48°44′96.76″	16°08′49.93″	237
			16.7.2016	3	Deer farm	Deer, wildlife	48°44′48.34″	16°07′48.31″	214
Oblekovice	Znojmo	Czech Rep.	16.7.2016	4	Sheep pasture	Sheep	48°50′16.96″	16°05′37.79″	246
Sobes, Podmoli	Znojmo	Czech Rep.	17.7., 29.7.2016	8 + 8	Organic vineyard	Wildlife	48°48′54.56″	15°58′36.05″	283
Uherčice	Znojmo	Czech Rep.	18.7.2016	5	Large cattle farm	Cattle (cats, dogs)	48°58′0.74″	16°39′54.00″	201
Nový Přerov	Břeclav	Czech Rep.	16.7.2016	5	Village house	Goats, horses	48°48′45.81″	16°30′11.39″	182
Rakvice	Břeclav	Czech Rep.	18.7.2016	5	Large cattle farm	Cattle (cats, dogs)	48°51′10.91″	16°48′41.10″	162
			18.7.2016	2	Horse farm	Horses, dogs, cats, sheep, goats	48°51′7.81″	16°48′50.33	161
Kurdějov	Břeclav	Czech Rep.	18.7.2016	2	Ecofarm	Horses, dogs, cats, goats	48°58′27.19″	16°45′29.31″	334
Březí	Břeclav	Czech Rep.	19.7.2016	5	Large cattle farm	Cattle (cats, dogs)	48°48′46.83″	16°34′23.69″	195
Kobylí	Břeclav	Czech Rep.	19.7.2016	5	Large cattle farm	Cattle (cats, dogs)	48°55′42.79″	16°54′07.75″	290
Velké Bílovice	Břeclav	Czech Rep.	19.7.2016	4	Large cattle farm	Cattle (cats, dogs, sheep, pigs)	48°50′44.07″	16°52′51.33″	187

The sand fly specimen was transferred to 70% ethanol, head and genitalia were slide-mounted using CMCP-9 mounting medium (Polysciences) and the rest of the body was stored in ethanol for molecular analysis. Morphological identification was carried out using published keys and descriptions [4, 10]. Identification was confirmed by a sequencing analysis of the cytochrome oxidase I (COI) gene. Genomic DNA was isolated with a High Pure PCR Template Preparation Kit (Roche). PCR amplification of COI was performed in a 25 µL reaction volume, using the LCO1490/HCO2198 primer pairs and amplification conditions previously described [7]. The amplification products were separated and visualised on 1% agarose gel, purified using a High Pure PCR Product Purification Kit (Roche) and directly sequenced in both directions using the primers used for DNA amplification (ABI Prism BigDye Terminator Cycle Sequencing Ready Reaction Kit). The new COI sequence of the *Ph. mascittii* specimen from Slovakia (length 620 bp) was deposited in GenBank (Accession Number KX963380). It was blasted against the GenBank database for identification and then aligned and compared with sequences of *Ph. mascittii* (KX869078, KX981913–KX981916) downloaded from GenBank.

## Results

**Figure 1.**
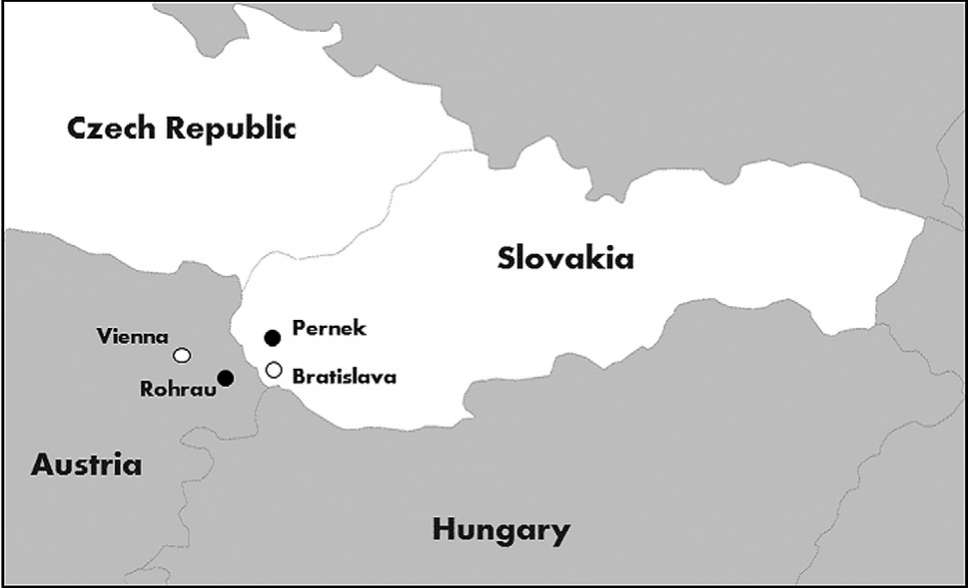
A map showing the location of Pernek with relation to the nearest previous record of *Ph. mascittii* in Rohrau, Austria.

**Figure 2.**
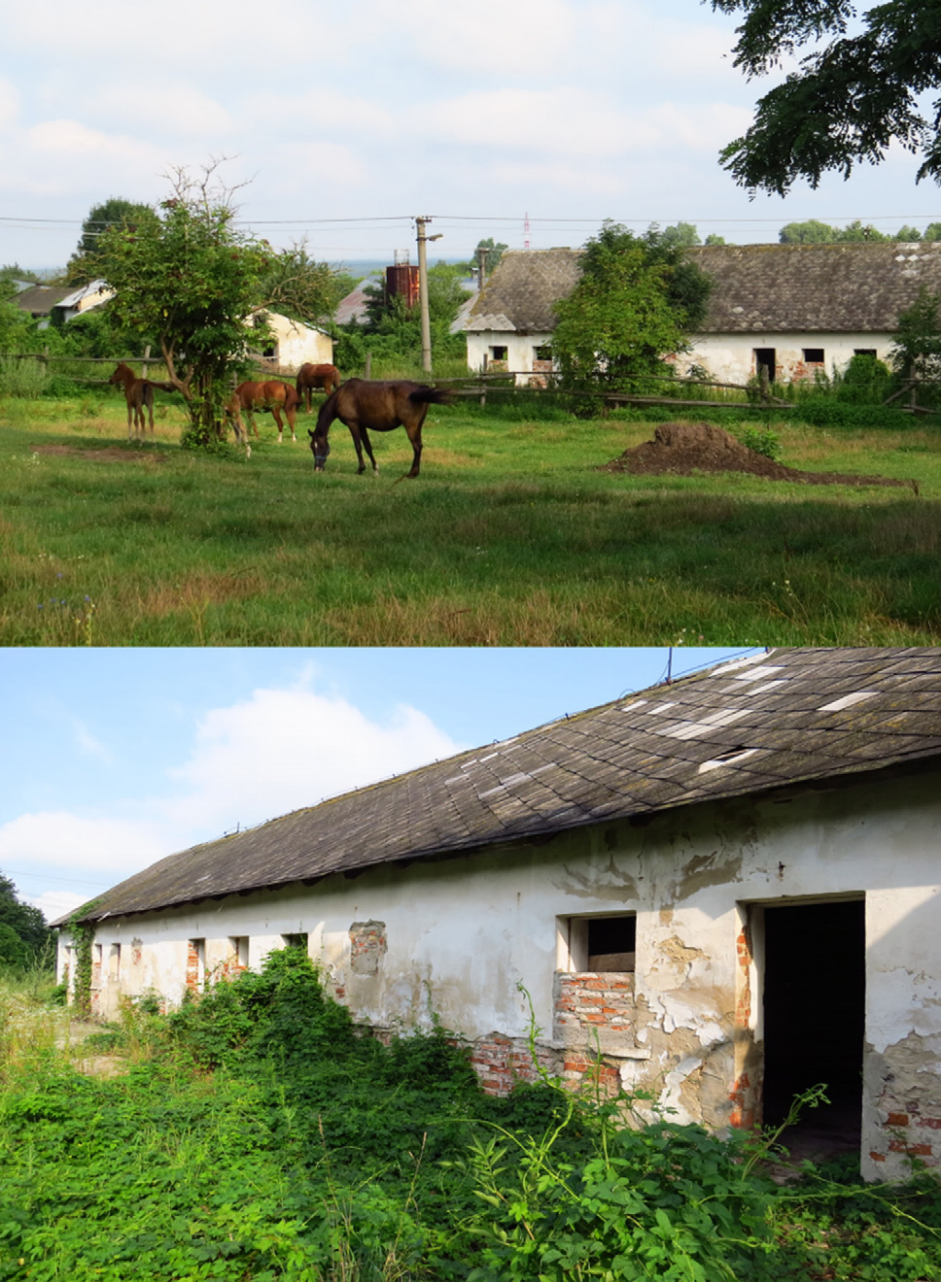
A barn on the farm in Pernek, Slovakia where the female *Phlebotomus mascittii* specimen was collected.

Inspection of insects collected in previous seasons in south-eastern Slovakia did not reveal the presence of sand flies. Out of 41 localities surveyed in summer 2016, a single female sand fly was found in one locality, namely Pernek in Slovakia. This village is situated at the western slope of the Small Carpathians, a low mountain range that forms a part of the Western Carpathians mountain system (Fig. 1). The sand fly was trapped in a partly disused barn on a former cattle farm where only about 25 horses are bred at present (Fig. 2).

**Figure 3.**
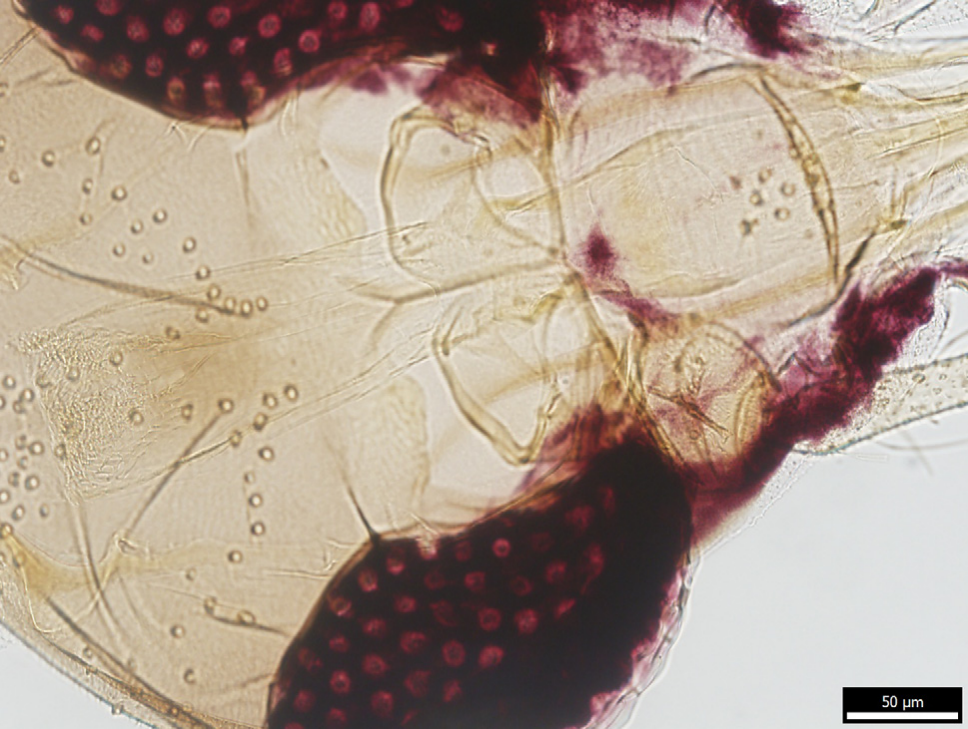
Pharynx of the examined *Ph. mascittii* specimen with typical pharyngeal armature.

The specimen was identified as a female *Phlebotomus mascittii* by traditional morphological characters of the pharynx (Fig. 3) and genitalia. The obtained part of the COI gene sequence (GenBank KX963380) was blasted against the GenBank database and identified as *Ph. mascittii*. A constructed alignment of the sequence of the Slovak specimen with the above-mentioned sequences of *Ph. mascittii* from Slovenia confirmed the GenBank identification and revealed only a single polymorphic site at position 106.

## Discussion

This study presents the first finding of phlebotomine sand fly *Phlebotomus mascittii* in Slovakia that adds to the several northernmost records of this species in Europe. The fact that it was this particular species is not surprising; it has been assumed that *Ph. mascittii* has a large range of distribution and it is present throughout most European countries of the Mediterranean basin [9] as well as adjacent areas north of this region, including sporadic findings in Belgium [4], Germany [13, 15], Austria [16] and Hungary [6]. A recent single record in Algeria also suggests its occurrence in North Africa [2]. Other species of the subgenus *Transphlebotomus* seem to have markedly more restricted distribution. However, a recent description of two new species of this subgenus, *Phlebotomus killicki* and *Ph. anatolicus* [8], raised the question of whether the widespread presence of *Ph. mascittii* may be partly due to these two previously unrecognised species and suggests that exact distribution of species within the genus *Transphlebotomus* has not yet been delineated unambiguously.

Our finding of *Ph. mascittii* in southern Slovakia confirms the presence of this species at the northern limit of subgenus *Transphlebotomus* distribution. This species was previously recorded in neighbouring countries Austria and Hungary. In Hungary, specimens of *Ph. mascittii* were sporadically recorded in Baranya county at the southern border with Croatia, in Veszprém county close to Lake Balaton and in Pest county in the suburbs of the capital Budapest in 2006–2009 [6]. The latter observation was supported theoretically by climate modelling, suggesting that the peri-urban environment at the outskirts of Budapest would be favourable for this species under certain scenarios [1]. Our survey, however, did not record any sand flies in areas close to the Slovak-Hungarian border. In Austria, *Ph. mascittii* was first recorded during entomological surveys in Carinthia (2009–2010), the southernmost region of the country neighbouring Slovenia [16] and thus very distant from our positive site in Slovakia. However, a more detailed survey in the following seasons (2012–2013) revealed small but stable populations of *Ph. mascittii* in localities in Styria, Burgenland and Lower Austria with the northernmost record in the village of Rohrau close to the capital Vienna and Austrian-Slovak borders [17]. This area, called Hundsheimer Berge, is in fact the southernmost extension of the Small Carpathians where our specimen of *Ph. mascittii* was collected. Future genetic comparison of Austrian and Slovak specimens should reveal whether they belong to one or two closely related populations. Interestingly, the specimen from Slovakia showed almost 100% identity with sequences of *P. mascittii* specimens from Slovenia in sequences of COI, which is a mitochondrial marker often used in molecular systematics of sand flies [3].

Our knowledge of the biology, ecology and epidemiological significance of *Transphlebotomus* species in the transmission cycles of leishmaniases is incomplete and sometimes contradictory: while some authors have speculated that *Ph. mascittii* is autogenous and hence not important for *Leishmania* transmission [4], others assume that this species readily feeds on dogs and humans and it has been proposed as a potential vector of *Leishmania infantum* in several small foci of presumably autochthonous canine leishmaniasis in Germany [15]. More importantly, an ITS1 (internal transcribed spacer 1) real-time PCR assay recently revealed one female positive for *L. infantum* DNA among ten tested ungorged females of *Ph. mascittii* caught in Austria [18]. However, experimental infections of this species have not yet been studied. It is also unresolved whether *Transphlebotomus* species share similar habitats with other sand fly species or inhabit special niches. While one of the newly described species, *Ph. anatolicus*, was collected in typical sand fly habitats near domestic animals [8], other *Transphlebotomus* species are represented in low numbers in usual sand fly surveys, and *Ph. mascittii* was recorded mainly from cavernicolous habitats [14]. The disused barn found positive in our study may simulate this type of habitat. Curiously, one Asian elephant (*Elephas maximus*) belonging to a commercial circus company was also kept close by, although a CDC trap which was placed near to it did not reveal any sand fly specimens.

Our single finding suggests that detailed entomological survey is needed to elucidate the extent of sand fly presence in the region of southern Slovakia, northern Austria and Hungary, as their eventual establishment may have implications concerning possible future transmission of canine or human leishmaniases.
